# Audience entrainment during live contemporary dance performance: physiological and cognitive measures

**DOI:** 10.3389/fnhum.2015.00179

**Published:** 2015-05-05

**Authors:** Asaf Bachrach, Yann Fontbonne, Coline Joufflineau, José Luis Ulloa

**Affiliations:** ^1^UMR 7023 CNRS/Université Paris 8Paris, France; ^2^Labex ARTS H2H Université Parsi 8Paris, France; ^3^UMR 8218, Université Paris 1 - Panthéon SorbonneParis, France

**Keywords:** entrainment, spectating, duration estimation, synchronization, contemporary dance

## Abstract

Perceiving and synchronizing to a piece of dance is a remarkable skill in humans. Research in this area is very recent and has been focused mainly on entrainment produced by regular rhythms. Here, we investigated entrainment effects on spectators perceiving a non-rhythmic and extremely slow performance issued from contemporary dance. More specifically, we studied the relationship between subjective experience and entrainment produced by perceiving this type of performance. We defined two types of entrainment. Physiological entrainment corresponded to cardiovascular and respiratory coordinated activities. Cognitive entrainment was evaluated through cognitive tasks that quantified time distortion. These effects were thought to reflect attunement of a participant’ internal temporal clock to the particularly slow pace of the danced movement. Each participant’ subjective experience—in the form of responses to questionnaires—were collected and correlated with cognitive and physiological entrainment. We observe: (a) a positive relationship between psychological entrainment and attention to breathing (their own one or that of dancers); and (b) a positive relationship between cognitive entrainment (reflected as an under-estimation of time following the performance) and attention to their own breathing, and attention to the muscles’ dancers. Overall, our results suggest a close relationship between attention to breathing and entrainment. This proof-of-concept pilot study was intended to prove the feasibility of a quantitative situated paradigm. This research is inscribed in a large-scale interdisciplinary project of dance spectating (labodanse.org).

## Introduction

### Dance and Spectating

Dance is a fundamental form of human expression that involves rhythmic or arrhythmic body movements, and often but not always is accompanied by music. It is performed alone or as a collective activity and is practiced in many cultures as a form of emotional expression, social interaction, or exercise. The kind of movements that can be considered as dance movements is difficult to establish in part due to the variety of dance styles that exist, each with their own historical and cultural specificities. Despite the differences, in all types of dance, the way the dancer moves seem to differ from the way we move in everyday life, sometimes in subtle manners. Similarly, watching dance might require a way to observe movements that is different from the way we observe movements in everyday life. In general, the attitude toward an artwork has been thought by some critics to evoke an openness, attentiveness and lucidity that allow a spectator to grasp without intention, but readiness, an artwork (Macel, 2008). Therefore, spectating is not simply sitting and being “moved” by the artwork. It is an active attitude. In addition, different persons may have different attitudes in relation to an artwork, which, in turn, may affect the way they perceive an artwork (Macel, 2008). In this scenario, spectating is both the specific ways we observe dance and the specific ways dance affects us. Here, we aimed at investigating how dance perception affects us, on cognitive and physiological dimensions, and how these modulations are related to our subjective experience.

The confluence of bodily movements, gestural expressions, and rhythm—whether observed by an audience or performed by dancers—is able to imbue the participants with feelings and emotions that compose the aesthetic experience of dance. Emotions are a fundamental aspect of many perceptual or cognitive states (Duncan and Barrett, 2007), and the experience of dance is clearly imprinted with emotions. In this context, a number of key questions about the experience of dance remain unanswered: What is the relation between the dancers’ experience and the spectators’ experience? Is there any relationship between the cognitive or physiological states experienced by the spectators and the one experienced by performers? Could the concomitant experience of dance induce analogous states between the dancers and the spectators, and is there any causal association between these states? Such challenging questions have just recently entered into consideration in cognitive neuroscience.

A hallmark aspect of dance, its rhythmical structure, has inspired a series of studies on dance production (e.g., Brown and Parsons, 2008). Less studied is the question of how such rhythms are perceived by, and affect the spectators. Furthermore, every dance piece or style contains multiple rhythms at different dimensions and granularities. Sometimes these rhythms can be clearly observed in the movement pattern or the music; they are explicit, but in other cases these rhythms emerge from the structure of the choreography or the state of the dancers (they could be considered as implicit). In contemporary dance, implicit rhythms are of particular interest since it is often the case that the music (if present at all) and the movement lack an explicit rhythmical structure. In order to study implicit rhythms we need to deepen our understanding of a dance specific choreographic structure, and to look at the individual dancer’s cognitive and physiological states underlying their quality of movement. Here, we will revisit the notions of mirroring and coupling of rhythms (or entrainment) from their sources in bodily movements and their neural representations. Next, we will propose a redefinition of entrainment that covers a wider range of phenomena beyond explicit rhythms, to include implicit rhythms (rhythms that are “under the skin”). We will use a unique choreographic style as our paradigm to investigate a specific case of rhythmic entrainment, and a multi-level methodology to study its physiological and cognitive aspects. Bearing in mind that a true understanding of dance spectating should take into consideration the subjective experience of dance, we elaborated a series of questions—in the form of a *post hoc* questionnaire—whose answers will guide the interpretation of our experimental findings.

### Movement, Feeling and Myriam Gourfink’s Work

At the heart of dance spectating is the observation of sophisticated bodily movements performed by dancers. Neuroscientists have studied extensively how action is realized in the nervous system and what are the effects of such representations in cognition and perception. Action representation has been shown to be multifaceted and to span multiple levels of complexity in the neuroaxis that increases from the muscle to the spine, and to the brain (Grafton and Hamilton, 2007). The multifaceted aspect of action has led investigators to consider that action and perception are two facets of the same phenomenon. Perception has been thought of as an implicit preparation to respond (Sperry, 1952). The view that action and perception are coupled has been in part supported by the discovery of mirror neurons in the monkey motor cortex that exhibit similar responses to the sight of a given action and to the execution of the same action (di Pellegrino et al., 1992; Rizzolatti et al., 1996). The regained interest in this topic has inspired further investigations of potential relationships between action and perception. The outcome of all these studies is that not only actions are represented in the nervous system, but also that these representations can be emulated covertly or overtly in a number of ways, including the observations of other’s movements (Jeannerod, 1999). Evidence supporting this conclusion comes from a variety of studies analyzing motor potentials (e.g., Fadiga et al., 1995) and motor-related brain activity (e.g., Stevens et al., 2000; Buccino et al., 2001). These studies show that the mere observation of other’s movements evokes(?) a specific and exquisite regulation of the motor nervous system in the observer. This evidence is extended to situations where we perform and observe complex sequences of movements, like in dance. In a study that involved dance experts of capoeira and ballet watching these distinct types of dances, Calvo-Merino et al. (2005) showed more engagement of action-related neural systems when dancers observed movements in which they have been trained. Cross et al. (2006) further extended these studies, showing that the involvement of action-related brain systems systematically builds up as dancers practice their movements along 5 weeks of training. Lastly, Jola et al. (2012) has shown that the experience of dance, merely as spectator, modulates specific motor responses when beholding a dance style to which the observer has been accustomed. Overall, these studies demonstrate that spectating a dance piece engages our own motor system.

Are the cognitive and emotional states that emerge during dance spectating mediated via these action simulation mechanisms? In the field of neuroaesthetics, the perception of artworks such as paintings was shown to involve activation of perceptual and affective brain systems (Jacobsen et al., 2006; Nadal et al., 2008; Chatterjee, 2011). In addition, some recent investigations have suggested that action neural systems are engaged in aesthetic judgments of artworks (e.g., Kawabata and Zeki, 2004; Cela-Conde et al., 2009). One of the notions suggested in this framework is that the artist’s actions could be captured in the artwork style, and thus elicit an embodied simulation response in the observer (Freedberg and Gallese, 2007). For example, observing the brush-strokes of a canvas could evoke the artist’s intentions through a motor simulation in the observer. Recent evidence gives support to these hypotheses. For instance, Leder et al. (2012) showed that active execution of movements increases the viewer’s liking rates when they match the style of the paintings.

The case of dance seems to be very well suited to evaluate these theories insofar as the art object contains live bodily movements. In fact, a study conducted by Calvo-Merino et al. (2008) analyzed brain activity while naïve participants evaluated short sequences of dance in distinct aesthetic dimensions. They showed that high ratings were associated with a stronger engagement of action-related neural systems. This and other studies indicate that the aesthetic experience of dance involves sensorimotor processing (arguably) of the gesture underlying the artwork (Calvo-Merino et al., 2008; Cross et al., 2011). In addition, one could argue that the aesthetic experience of the spectator could be associated with explicit emotional responses. In fact, dance gestures and movements can be explicitly expressive and thus could evoke emotional responses in the public (Atkinson et al., 2004). However, in many cases in contemporary dance, the aesthetic experience can be derived neither from explicit emotional expressions nor the production of recognizable gestures, since the dance work can eschew both elements. In these conditions the dance experience could emerge from the “infusion” or “permeation” of physiological or cognitive states between the dancers and the public (Haggard, 2012). In order to inform our research on spectating, we take into account studies in the field of humanities and performance, which by focusing on the uniqueness of each artistic proposal and the spectator attitude, examine the multiplicity and complexity of this experience at several dimensions: the visual part, the kinesthetic part, as well as at different levels: sensory, emotional, imaginative and interpretive. The studies presented here, and the larger research program which they are part of, are an attempt to build bridges between the humanities and the cognitive sciences. The approach consists in taking into consideration both the aesthetic and phenomenological aspects of spectating when setting-up experimental protocols. We believe this kind of bi-directional bridge has the potential to enrich both quantitative research common to the cognitive sciences and qualitative research practiced in dance studies. While the phenomenological perspective allows for a more nuanced and rich modeling of the subjective experience, experimental data can help clarify some of the neurophysiological mechanisms at play. Here, we investigated changes in the cognitive and physiological states of participants spectating a distinctive dance style, that of the French choreographer Myriam Gourfink.

The choreographer Myriam Gourfink has developed a specific speed and quality of movement that make her style unique and recognizable beyond the specificities of each piece (for internet examples see: www.myriam-gourfink.com/deperdition.html and www.myriam-gourfink.com/uneLenteMastication.html). Dancers can spend 8 min to cross 10 cm (Lesauvage and Piettre, 2011). Gourfink’s style is characterized by a movement speed far below these regular bodily movements such as regular walking, tapping or clapping (with a frequency between 120 and 130 bpm). Gourfink’s continuous extremely slow movement (with no change in the rhythm) doesn’t consist in slowing down or reproducing one movement more slowly. It is induced by a technique that changes brain and body states: “energy yoga”. According to the choreographer [it] is the breathing that entrains this slowing-down of movement (Lesauvage and Piettre, 2011). The fundamental aspect of energy yoga is the generation of slow movements and controlled breathing (Lesauvage and Piettre, 2011). During the energy yoga sessions and during the performance, dancers share their attention between the respiration, micro-movement and body sensations (Lesauvage and Piettre, 2011). Energy yoga is at the root of the company’s training: at each rehearsal, the dancers and the choreographer practice this yoga-meditation during up to 5 h before beginning the dance practice. Every live performance is also preceded by a few hours of yoga practice that brings about the characteristic quality of presence and speed of movement. In addition, neither mimicry nor theatralized expressions can interrupt the spectator’s experience. The hallmark of Myriam Gourfink’s work is a slowness of movement and an intimately associated slowness of respiratory rate. This extremely slow movement challenges the spectator’s perceptive systems. One often experiences blindness to the progressive postural changes in the visual scene. In addition, some spectators report a strange experience of space and time just after the presentation and are surprised by the objective length of the performance that they often underestimate. The effects that Myriam Gourfink’s choreography produces on spectators have been subject to many studies in the field of performance, phenomenology and aesthetics. In general, theses studies show that spectators can “lose spatiotemporal marks” (Fontaine, 2004, p.137) and become “aware of her/his perceptual experience” moment to moment (Gioffredi, 2008). One major effect of her choreography is an increased bodily self-consciousness and kinesthetic sensation. The studies presented here (and the labodanse project) represent a first attempt to bring together theoretical and experimental methods from humanities and the cognitive sciences to study the experience of spectators of Myriam Gourfink’s work, with the more general intention to build up an integrated view of dance spectating. These studies are highly informed by the qualitative literature that shapes our hypothesis space and at the same time allow us to explore (quantitatively) relationships between different dimensions of the subjective experience and between subjective experience and (intersubjective) physiological factors.

### Beyond Explict Rhythms

One key element of dance—present as well in Myriam Gourfink’s work—is its dynamic inter-subjective aspect. This implies a co-presence of bodies and a real time relationship between the experience of the spectators and the one of the performers. An emergent idea in cognitive neuroscience is that coordinated behavior between two or more persons is a fundamental aspect of human interaction (Knoblich and Sebanz, 2008). Coordinated behavior can be achieved via the attunement of a person’s rhythms to the rhythms of another person, an entrainment. The notion of entrainment has been borrowed from physics to indicate the phenomenon by which two rhythmic processes interact with each other so that they adjust themselves and eventually become rhythmically coupled (like bearing the same phase; Clayton, 2012). This notion was extended beyond physics to include many natural and cultural phenomena that have a periodic nature. Examples of such phenomena include the synchronous activity of neuronal groups, the synchronous social behavior in animals and cultural phenomena such as dance. Since humans coordinate their activities in a variety of events during daily life, this coordinated behavior has been thought to be important in social interactions, and has been said to promote cooperation (van Baaren et al., 2004). Coordinated motor responses seem to arise even in the absence of any explicit instructions to do so. Richardson et al. (2007) studied interpersonal coordination between two people sitting side-by-side in rocking chairs. They observed that people unintentionally synchronized, and that the strength of such coupling relied upon information that the participants have of each other. Entrainment has been studied also in relation to musical action synchronization (Clayton, 2012) as well as synchronization between two or more dancers performing a rhythmically structured movement (see the current issue). Here, we propose to enlarge the notion of entrainment to incorporate a wider spectrum of phenomena, and include the dynamic coordination that happens in the absence of an explicit rhythmical structure, like in many forms of contemporary dance. This may allow us to measure coordinated behavior that may not bear strict oscillatory features. We suggest that audience entrainment could be measured by assessing the coordination of physiological activities between performers and spectators at multiple levels: autonomic, kinesthetic, and neural. In our study, we aimed at investigating entrainment by inspecting two kinds of rhythms that could be particularly modulated by Myriam Gourfink’s work: the respiratory rate and the internal temporal clock.

The respiratory rate is a signal of the autonomic nervous system, thought to echo emotional states and responses (Boiten et al., 1994). Autonomic signals such as the respiratory rate, and others such as cardiac responses, have also been studied in the context of real-time inter-subjective engagement. For instance, certain studies have assessed coordination of autonomic signals between pairs. Respiratory and cardiac responses have been shown to be coordinated between romantic partners (Helm et al., 2012). In addition, researchers have found, in a more natural setting, a co-variation of cardiac responses between participants and spectators in a collective ritual (Konvalinka et al., 2011). More recently, other studies have investigated collective vs. individual behavior and have shown coordination of autonomic signals, in this case, respiratory responses, even in the absence of synchronized actions (Codrons et al., 2014). Since respiration is a fundamental aspect of Myriam Gourfink’s work and respiration synchronization has been shown to be associated with intersubjective coordination, we set up a first experiment to explore this topic. In this experiment we analyze whether synchronous respiratory rates between spectators and performers were correlated with subjective reports (in particular, those reflecting attention to respiration).

The experience of time has been thought by some investigators to be dependent on an internal clock mechanism or pacemaker (Hoagland, 1933). In this model pulses are accumulated, stored in working memory, and then compared to a reference (Gibbon et al., 1984). The estimation of time has been shown to be modulated by a complex ensemble of brain-body factors that include cognitive, emotional and physical states (Wittmann and van Wassenhove, 2009) and by the characteristics of the stimulus we perceive (Nather et al., 2011). Attention and arousal have a decisive role in time perception. An increase of arousal induced by emotions like fear (Grommet et al., 2011), or induced by physical activities, bring about temporal over-estimation (Cahoon, 1969). On the other hand, altered states of consciousness induced by “brain-body techniques” such as hypnosis (Schwartz, 1978) and mindfulness meditation (Berkovich-Ohana et al., 2011) have been reported to induce temporal under-estimation. These states are associated with an increase of attention (particularly internalized) and reduced arousal. Interoceptive focus has been shown to increase time distortion in both senses: over and under estimation (Pollatos et al., 2014). Studies show that temporal over-estimation can be induced by static pictures depicting distinct body postures (Nather et al., 2011; Orgs et al., 2011), or video stimuli portraying dance movements displayed at different speeds (Sgouramani and Vatakis, 2014), suggesting thus, that modulations of the speed of an observed (or implied) body movements might have an effect on the perception of time. As discussed earlier, extremely slow movements are fundamental in Myriam Gourfink’s work. According to aesthetic and phenomenological studies a major effect of this choreography on the spectator is an increased awareness of movement, millimeter per millimeter, a changed temporal awareness associated with an increased interoceptive focus, and an increased kinesthetic sensation (Gioffredi, 2008). In this sense, studies in aesthetics guided our experimental work for the construction of a *post hoc* questionnaire. As in studies focusing on meditation—which investigate the relationship between time perception, attention regulation and body awareness in a “static posture” (Sauer et al., 2012; Kramer et al., 2013; Wittmann et al., 2015)—Myriam Gourfink’s work is also an interesting way to explore the relationship between time perception and bodily awareness as well as movement (and its perception). In our second experiment we analyzed whether time distortion effects in the spectators were correlated with their subjective reports (engagement, attention to the movement and interoceptive awareness).

Our investigation is inscribed in the approach to human interaction as a dynamic phenomenon that could be better understood using real-life paradigms. Instead of using video stimuli we use paradigms where spectators attend to a real performance in a theatre or attend to a semi-controlled experimental setting (spectators observe live extracts of the full performance in a dance studio).We think that this methodology has the potential to provide a better insight into how dance performance affects spectators. Video presentations do not always produce the same neuro-kinaesthetic effects that a live performance does (Jola and Grosbras, 2013). In the spirit of the neurophenomenological approach (e.g., Varela, 1996) we elaborated a series of questionnaires and a multilevel approach to combine first and third person perspectives. For this study, we chose four questions that allow us to investigate the emotional engagement, kinesthetic attention and interoception degree or rate of the spectators. In this pilot study, we aimed at elaborating an integrative experimental approach that allows us to understand dance spectating. Further experimentation and improvement of our approach will be necessary to draw more definite conclusions.

## Methods

### Respiratory Entrainment and Subjective Engagement

We investigated whether respiratory entrainment was associated with the subjective engagement of the spectators. First, we measured the breathing patterns from both the dancers and the spectators to investigate respiratory entrainment. Next, we evaluated if the strength of the entrainment was correlated with the subjective experience of the spectators which was estimated using post-performance questionnaires (section Questionnaire). We performed this experiment in a semi-naturalistic setting where participants were invited to watch excerpts of Gourfink’s choreography in a dance studio. The experimental sessions were performed without music.

#### Subjects

We collected 28 measures in total. The measures were recorded separately over 4 sessions, where 7 audience members participated each time. From the total of participants 4 participated twice. Spectators (20 females, 4 males, mean age = 32 years, SD = 6.93) have all, but one, dance experience (13.3 years on average). The dancers were from Myriam Gourfink’s company and performed in all sessions. All participants provided written informed consent according to institutional guidelines of the local research ethics committee (in compliance with the Declaration of Helsinki) and were compensated for their participation.

#### Procedure

The experiment consisted in 4 experimental sessions (Centre National de la Danse, Pantin). In each session 4 excerpts of Gourfink’s choreography were presented. Upon arrival, spectators filled a general information questionnaire and were fitted with the Bioharness 3 sensor (a chest belt, Bioharness 3, Zephyr, USA). Breathing rate was collected using a mechanical breathing rate sensor embedded in the belt. The audience was then led into the dance studio and was seated in the first two rows of the tribune. The dance performance lasted about 35 min and was composed of 4 duets, each lasting about 7 min. Two dancers performed on each excerpt. The order of the duets was pseudo-randomized across sessions. After the dance performance, the audience completed a subjective appreciation questionnaire.

#### Data Processing

Breathing rate data was recorded at a sampling rate of 1 Hz. Data was filtered using a median filter (a 7th order low pass filter with a 20 sample point window). Next, the re-sampled data was z-normalized (per subject) using a pre-performance interval of 10 min as baseline. The data was synchronized across subjects using the markers (timestamp) provided by the Bioharness sensor. We then segmented the breathing rates time-courses into 4 time intervals corresponding to the 4 duets.

#### Analysis

Individual level analysis: For each spectator, we fitted the data to four (one per duet) multi-linear regression models using MATLAB R2007b (The MathWorks, Inc., Natick, Massachusetts, USA). The regression equation was YBR(t) = aX1BR(t) + bX2BR(t). YBR is the breathing rate of the spectator during the performance of the duet. X1BR and X2BR are the breathing rates of the two dancers from the duet. To study the combined effect of the two dancers’ breathing rate we extracted the model’s R2 (amount of variance explained by the model) rather than the coefficients of the individual dancers. In order to estimate the overall effect of the 4 duets we then averaged the four R2 values. We shall name this measure the R2 score.

Group level analysis: Individual responses to the four target questions were modeled using linear mixed models (lm4 package for R; Bates et al., 2014) with “session” and “R2 score” as fixed effects and “subject” as random effect. A linear mixed model was used rather than a simple correlation test since 4 subjects participated twice (see above).

### Time Perception and Subjective Engagement

Time perception is often quantified through tasks that demand the subject to estimate the duration of a stimulus presentation. We used a time perception task based on Lamotte et al. (2012)’s protocol to investigate the effects of Gourfink’s choreography on the estimation of time by the spectators before and after the performance in order to estimate changes in their internal clock. Next, we evaluated if the scores of the individual changes were correlated with the subjective experience of the spectators which was estimated using *post hoc* questionnaires (section Questionnaire).

#### Subjects

Twelve participants were recruited at the theater Lobby near Paris prior to the live performance of Myriam Gourfink’s company. All particpants provided written informed consent according to institutional guidelines of the local research ethics committee (in compliance with the Declaration of Helsinki) prior to inclusion in the study. They were not paid for participation. Two groups of participants (*n* = 5 and *n* = 7) completed the task before and after the dance performance on two different evenings.

#### Stimulus

The experiment was performed in the context of a live dance performance (Souterrain; for an internet example see http://www.myriam-gourfink.com/souterrain.html) with 10 dancers at the “Forum du Blanc-Mesnil” theatre. For the time perception task the stimulus to be timed was a blue square (1000 × 1000 mm) presented in the center of the computer screen on a black background.

#### Procedure

Before the start of the live dance performance, participants were received in a quiet section of the theater Lobby. The participants were seated in front a computer that controlled the experimental events and recorded the responses via the Open Sesame software (version 0.26). In the task, the participants had to judge the stimulus duration in milliseconds and give their responses on a numeric keypad. They were instructed that the stimulus duration was between 200 and 1800 ms. When the participants were ready, they started a trial by pressing the spacebar. A blank screen was presented for 500 ms and then a rectangle appeared. There were 6 stimulus durations: 400, 600, 800, 1000, 1200, and 1400 ms. A 500 ms blank screen followed the presentation of the rectangle and then a screen displaying a request for time estimation. After the participants gave their response they had to press the spacebar to begin a new trial. Before the actual experiment participants went through a training session that started with a presentation of a single, labeled example of a 1000 ms duration, followed by 12 practice trials (of the same structure as the actual experiments) but with only 200 ms and 1600 ms durations. After training the participants performed the experiment. They completed 6 trials per each of the 6 types of presentation durations (36 total trials). After the live dance performance participants completed an identical task (without the practice block). After completing this task participants filled a questionnaire regarding their appreciation and subjective experience during the live dance performance.

#### Analysis

Our aim was to evaluate whether changes in temporal perception (indexed as the difference in subjective duration estimation before and after the performance) were correlated with changes in the degree of individual engagement and attention assessed through the post-performance questions. First we calculated the precision ratio (PR) as the difference between the subject’s estimation and the actual presentation length, divided by the presentation length (Lamotte et al., 2012), for each trial before and after the dance performance. A negative precision value indicates an underestimation of the stimulus duration. Next, we computed the temporal precision change (TPC) of a subject as the difference of PR between these two sessions. This reflects the subject’s temporal estimation changes as a consequence of beholding the dance performance. A negative TPC indicates that observing the live dance performance produces a contraction in the perception of time. Individual TPCs were calculated by taking the PRs, per trial, per subject, and entering them into a linear mixed model analysis with “session” (before/after the performance) and “date” (given that we performed the experiments on two different nights) as fixed effect and “subject” and “session” as random effects. We then extracted the model’s estimate for each subject’s (random) “session” effect, and added it to the fixed “session” effect estimate. The use of a mixed model to estimate individual level effects has been shown to increase the accuracy of the estimates (Efron and Morris, 1977). Individual TPC scores were then correlated with the individual responses to the 4 selected questions from the questionnaire.

#### Questionnaire

In both experiments, participants responded to a set of closed and open questions regarding their experience during the live dance performance. The closed questions were statements (e.g., “I enjoyed this piece”) which participants were asked to evaluate on a 5-point scale (1 = fully disagree, 2 = disagree, 3 = neutral, 4 = agree, 5 = fully agree). The statements that we used for the analysis were: “I liked the performance”, “I paid attention to the dancer’s breath”, “I paid attention to my own breathing” and “I paid attention to the dancers’ muscular tension”.

## Results

### Respiratory Entrainment and Subjective Engagement

**Figure 1:**
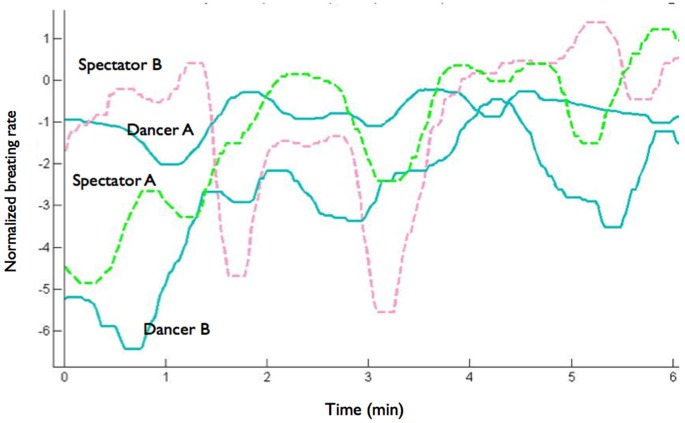
**Time course of the breathing rates for two spectators and two dancers during a solo dance presentation**.

As an illustration of the respiratory entrainment we plot the time course of the breathing rates for two spectators and two dancers during a dance presentation (Figure 1). Visual inspection suggests that the breathing rate of Spectator A is more attuned to the breathing of the dancers than the breathing rate of the spectator B. The R2 of the associated model for spectator A is indeed higher than that of the model for spectator B (0.6331 vs. 0.0263). The mean R2 score across subjects was 1.87 ± 0.063 (M ± SD).

**Figure 2:**
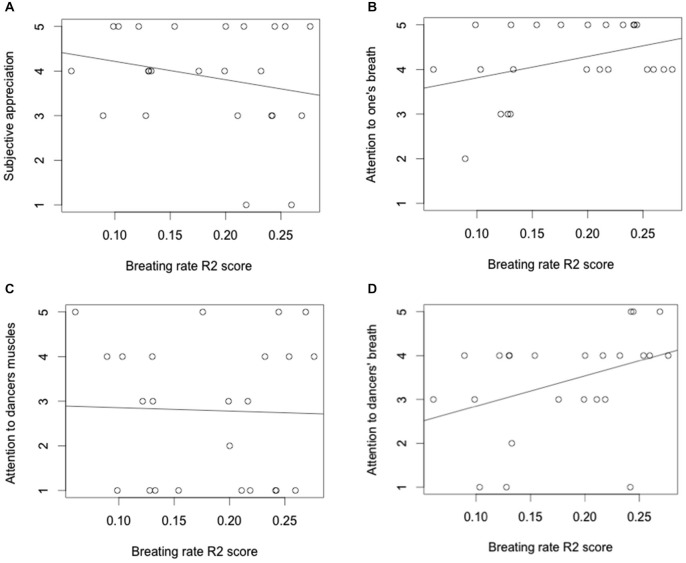
**Relationship between the subjective responses of the public to the questions regarding subjective appreciation (A); attention to one’s breath (B); attention to the dancers’ muscles (C) and attention to the dancers’ breathing (D), and the synchronization of their breathing rates with that of the dancers (reflected in the R2 scores)**.

We modeled the individual responses to the questions with the R2 breathing scores. The regression analyses (Table 1) showed a significant effect of ratings for two of these questions; one related with attention to one’s breathing and the other related with attention to the dancers’ breathing. We observed that the more attention a participant pays to her/his own breathing, the higher was her/his R2 score (indicating higher synchronization or entrainment, Figure 2B). In addition, the more attention a participant paid to the dancers’ breathing, the higher was his/her R2 score (indicating higher synchronization or entrainment, Figure 2D). The correlations between breathing synchronization and the other two questions were non-significant (Figures 2A,C).

**Table 1:** **Relationship between the subjective responses from the public and the synchronization of their breathing rates with that of the dancers (reflected in the R2 scores)**.

Question	Rating (M ± SD)	estimated effect of R2	*T*-value	*p*- value
“I liked the piece”	4.39 ± 1.19	−1.34	−0.53	> 0.1
“Often, I paid attention to my breathing”	4.24 ± 0.83	7.73	4.51	< 0.001
“I paid attention to the dancers’ muscle tension”	2.88 ± 1.62	−0.82	−0.25	> 0.1
“I paid attention to the dancers’ breathing”	3.44 ± 1.15	9.15	3.98	< 0.001

*M: average; SD: standard deviation*.

We evaluated the effect of perceiving the performance on the participant’s estimation of time. We did not observe a significant effect of session on the precision ratio at the group level (*t* = 0.802, *p* = 0.436), but we note that the coefficient was negative (*β* = −1.35607).

### Time Perception and Subjective Engagement

**Figure 3:**
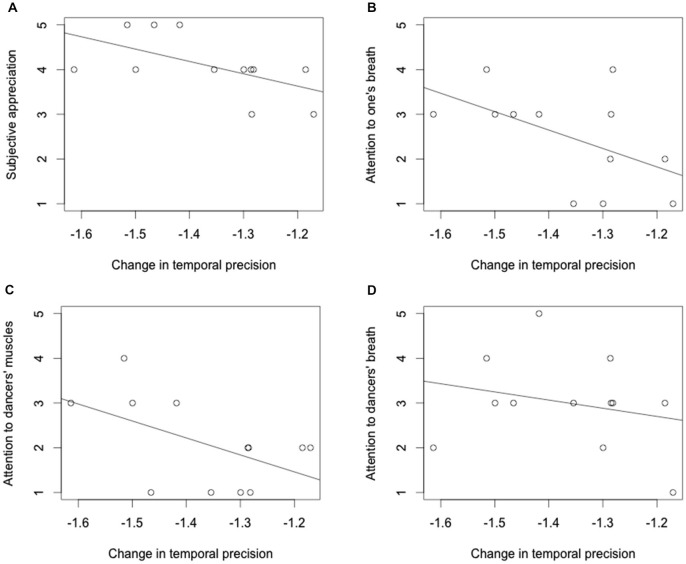
**Relationship between the subjective responses from the public to the questions regarding the subjective appreciation (A); attention to one’s breath (B); attention to the dancers’ muscles (C) and attention to the dancers’ breathing (D), and the estimation of time (reflected in the PRC scores)**.

We correlated the individual TPC scores with the individual responses to the questions. The correlation analyses (Table 2) showed a significant effect of ratings for three of the questions; one related with attention to one’s breathing, other related with attention to the dancers’ breathing and other related with attention to the dancers’ muscles tension. We observed that the more attention the participant pay to their own breathing, the more negative their temporal precision change (TPC) is, reflecting increased under-estimation of time (Figure 3B). In addition, the more attention the participant pays to the dancer’s muscles tension, the more negative his/her TPC is (Figure 3C). Finally, we found a marginally significant negative correlation between the degree to which a participant enjoyed the performance his/her TPC is (Figure 3A). The correlation between TPC and the question regarding attention to the dancer’s breath was non-significant (Figure 3D).

**Table 2:** **Relationship between the subjective responses from the public and the scores of estimation of time**.

Question	Rating (M ± SD)	Pearson’s correlation	*p*-value
“I liked the piece”	4.08 ± 0.67	−0.57	0.0527
“Often, I paid attention to my breathing”	2.50 ± 1.00	−0.52	<0.05
“I paid attention to the dancers’ muscle tension”	2.08 ± 1.00	−0.53	<0.05
“I paid attention to the dancers’ breathing”	3.00 ± 1.04	−0.24	>0.1

*M: average; SD: standard deviation*.

## Discussion

Our aim was to conduct a proof-of-concept study. We investigated modulations of physiological and cognitive states induced when spectators behold a dance performance and if they were correlated with their subjective experience of spectating. Our specific hypotheses were guided by crossing phenomenological research on Myriam Gourfink’s work, and cognitive science literature on related questions. First, in a semi-live setting we investigated whether synchronization of respiratory rates between the dancers and the public during the performance is associated with the subjective experience of the spectators. We found that individual synchronization scores (how well the breathing rate of the dancers explains the breathing rate of the spectators) were correlated with the spectator’s subjective experience, specifically regarding how much the spectator paid attention to his own breathing and the breathing of the dancers. Second, in the context of a full production live performance we investigated whether changes in the temporal perception of the spectators were associated with their subjective experience during the performance. We found that the extent of time distortion effects (after vs. before the performance) correlated with the degree to which participants paid attention to the dancer’s muscular activity, and paid attention to their own breathing. In addition we found a marginal correlation between change in temporal perception and subjective appreciation of the piece.

### Respiratory Entrainment and Subjective Engagement

We investigated to what extent the subjective experience of spectators observing excerpts of Myriam Gourfink choreography is associated with the strength of their breathing entrainment by the dancers. We found that the strength of breathing entrainment (R2 score) had a significant effect (in a mixed linear regression model) on the spectator’s responses regarding attention to the dancers’ breathing (effect size = 9.14) and their own breathing (effect size = 7.72). Our results suggest that the degree of synchronization of the participant’s breathing with that of the dancers is associated with the spectators’ attention to breathing. This result illustrates the role of the participant’s attention to breathing in synchronous phenomena. Breath is a fundamental aspect of Myriam Gourfink’s work, underlying the specific speed and quality of the dancers’ movement. It seems plausible to speculate that the choreography strengthens attention toward breathing, thus producing breathing entrainment in the spectators. However, our methodology does not allow us to infer any directionality; is it the increased attention that brings about synchronization or is it the other way around? Or maybe there is a third factor? In our experiments the spectator’s attention was not guided during the performance (the questions were asked post-performance). One may also wonder whether guiding the spectator’s attention (for example to focus on the breathing of the dancer) will produce an increase in breathing entrainment. This can be a subject of a following study that will compare the effects of instructing the spectators to focus on distinct elements of the performance or the dancers. Given that the dancers themselves are highly focused on their breathing, a third interpretation of our results could be in terms of joint attention (not directly tested here). Could joint attention and synchronization be one and the same phenomenon? Future studies will be conducted to address these questions. Despite the role of breathing in Gourfink’s work and the tight link between synchrony and inter-subjective engagement (e.g., Konvalinka et al., 2011), we did not observe a reliable relationship between respiratory entrainment and subjects’ responses regarding their appreciation of the dance or their attention to the dancer’s muscles. It is possible that changes in the appreciation of the dance are more difficult to evaluate than other questions in *post hoc* questionnaires. In this vein, in future experiments, we could envisage to perform measures of skin conductivity to disentangle distinct aspects of emotional engagement, such as arousal (Rickard, 2004; Latulipe et al., 2011). It is also possible that breathing entrainment is independent from other aspects of movement. For example, in a recent study that investigates synchronization of breathing within a group, Codrons et al. (2014) found that breathing synchronization occurs in the absence of movement synchronization. In future experiments we could consider to analyze covert motor simulation from the spectators by recording electromyographic activity of their muscles. In the same vein, it is possible that breathing synchronization does not play a role in spectator’s overall enjoyment during a dance piece (by Gourfink). In future questionnaires we will attempt to break down the notion of enjoyment or pleasure into more specific components.

### Time Perception and Subjective Engagement

Previous studies have established that altered states of consciousness can elicit modulations in the perception of time (e.g., Wittmann and van Wassenhove, 2009). In addition, although there is evidence of modulations of time perception by merely observing others’ bodies (e.g., Sgouramani and Vatakis, 2014), so far, only a handful of studies have investigated how time perception can be modulated by perceiving slow biological motion, or viewing a person in an altered state of consciousness. Here, we hypothesized that entrainment by the extremely slow rhythm of Myriam Gourfink’s work and/or the dancer’s “slow” state of body-mind will produce a slowing down of the spectator’s internal clock (reflected in time under-estimation) and that individual differences in spectating will correlate with the extent of this entrainment. We observed no differences between the estimation of time before and after spectating the performance (*p* = 0.436, *t* = 0.802) at the group level. However, an effect of underestimation of time after the performance is in some way suggested by the negative value of the estimation coefficient (*β* = −1.36). This will be verified in a follow-up experiment where more participants will be included.

In order to disentangle individual effects we correlated the time distortion scores for each subject with their agreement scores to four post-performance statements. We observed a marginally significant correlation of time distortion with the response regarding the liking of the performance (*r* = −0.57, *p* = 0.052). That is, the more a spectator liked the dance piece the stronger was the under-estimation effect following the performance. A correlation was also observed for the responses regarding paying attention to the dancers’ muscular activity (*r* = −0.53, *p* < 0.05). That is, the more subjects were attentive to the moving bodies of the dancer, the stronger was the following temporal under-estimation. Similarly the responses regarding paying attention to one’s own breathing were correlated with time under-estimation (*r* = −0.52, *p* < 0.05). All in all, our results suggest that spectators who were more engaged with the dance piece, who were kinesthetically attentive to the dancer’s body movements, and who paid attention to their own breathing, experienced a temporal contraction effect. We suggest that the slowing down of the internal clock (an implicit rhythm), experienced as a contraction of objective time, is due to spectating Myriam Gourfink’s particular choreographic style. We interpret this effect as an entrainment, that is, an attunement to the particularly slow rhythm of the choreography and/or the dancers’ state. Importantly, this is only one possible interpretation of our results. Another, slightly different, interpretation can be that the slow movement of the dancer has a general effect of bringing down physiological arousal for everybody but that the strength of the effect depends of the level of the intercoptive focus of the spectator (attention to respiration). On the one hand, a number of recent studies have demonstrated an effect of observed slow movement (or implied low arousal body state) on temporal perception (Nather et al., 2011; Orgs et al., 2011; Sgouramani and Vatakis, 2014). It is worthwhile to note three important differences with the current work. First, here we used live rather than recorded images; second, our results establish a relationship between individual qualities of attention and the effect on temporal perception (to which we return below). Finally, these other studies quantified the effect of perceiving slow movement on the temporal perception of that same event or time interval, while here we quantified the effect of spectating (a very slow movement) on temporal perception of an independent event, presented after the experience of slow movement (demonstrating a lasting altered state). A recent study by Pollatos et al. (2014) showed that an “introspective focus” condition (paying attention to the body and bodily reactions that might occur) strengthened time distortion more than an “exteroceptive focus” condition (carefully attending to visual details). So further experiments, including measuring arousal and giving an instruction to the participants regarding interoceptive or exteroceptive focus are required in order to asses the role of hypo arousal and that of interoceptive focus in temporal distortion after spectating.

Our study brings about more specific questions regarding the relationship between temporal distortion and specific subjective dimensions of spectating. The correlation between the contraction effect and increased attention to the body of the dancers suggests that at least part of this effect could be mediated via kinesthetic resonance. The relation between contraction effect and increased internally oriented attention is reminiscent of temporal contraction effects following certain forms of meditation (Glicksohn, 2001; Berkovich-Ohana et al., 2013) such as energy yoga. We speculate that the relationship between internally oriented attention and temporal contraction is observed when certain spectators attain a state of mind similar to the one of the dancers. In future studies we will look at dissecting which mechanisms allow this sharing of body-mind states. The lack of correlation between the spectators’ attention to the dancers’ breathing and changes in temporal perception contrasts with the results of the first experiment. Given the differences in the testing conditions it is hard to draw any conclusions from these differences.

### General Discussion

In cognitive neuroscience, entrainment has been proposed as a notion that could help in the understanding of dynamic intersubjective engagement. Here, we focus our study on a dance style whose observation could have particular physiological and cognitive effects on the spectator. We proposed two notions of entrainment, one physiological and another cognitive, which were quantified accordingly. We selected specific questions to investigate distinct aspects of the subjective experience of the spectators during the performance: her/his emotional engagement (question 1); attention to the dancer’s muscles (2); attention to the dancer’s breathing (3) and attention to her/his own breathing (question 4). We found both types of entrainment to be related to the subjective responses of the spectators. A remaining, intriguing question is the relationship between these two types of entrainment. Does breathing synchronization play a role in the slowing of the internal clock? To address this question we are currently running experiments in which both dimensions are quantified concomitantly. Our subjective measure was based on *post hoc* reports, and this might be a limitation in this study. We are now implementing an on-line method to capture the dynamically unfolding subjective experience during performance, and correlate it with our physiological measures.

Above and beyond the interpretation of our specific findings, this study demonstrates the feasibility of, and challenges faced by, a quantitative, situated methodology to study physiological and cognitive changes during dance spectating and the importance of a phenomenologically informed analysis of these changes.

A variable that we did not controlled was gender. As pointed by a reviewer, there is evidence that gender has an effect on spectating on both subjective and physiological levels (Hanna, 1988; Calvo-Merino et al., 2006; Hugill et al., 2009). This issue was beyond the scope of our study but could be of interest in future research.

In this study we made use of the specific style of Myriam Gourfink’s dance but did not address the question of the specificity of her style to the experience of spectating. One natural extension of this work will be to investigate to what extent entrainment can also be found in cases of other dance styles. In addition, our approach poses a number on non-trivial challenges which our study could address only partially. One major question is that of a control condition. Control conditions are often included in laboratory experiments in order to allow researchers to isolate the specific dimension they want to study. It is not trivial, on both conceptual and practical grounds, to establish definitive control conditions when studying spectating in an ecological paradigm. Given the multiplicity of factors that constitute a specific dance event and the relationship between them, how does one go about to construct an event that is similar on all dimensions but one? Can or should this eventual control condition still be considered as an ecological event? When, as in experiment 2, the study takes place in conjunction with a full production, the cost and logistics of creating a control condition are prohibitive. It is however important to keep in mind that in the studies reported here the issue of control is not central since we were not primarily interested in testing the specificity of Gourfink’s work but in evaluating the relationship between first- and third-person dimensions of a spectating experience.

On the epistemological side, qualitative research in humanities guided our experimental research. The quantitative approach can help us to understand the relationship between different dimensions of spectating observed in phenomenological and aesthetic studies of Myriam Gourfink’s work (for example between the temporal and body awareness effects). Our approach and results, in the tradition of the neurophenomological perspective, highlight the importance and potential of a constant exchange between (third-person) experimental cognitive science, qualitative research in the humanities, and (first-person) “practice as research” experience. Experimental science is not conceived as a way to validate qualitative research nor is it suggested that subjective experience can or should be reduced to observable neurophysiological data. Instead, concepts, methods and findings from these different fields can, and should be, mutually informative and possibly interwoven in an inter-disciplinary porosity that will positively challenge and enrich research in these different perspectives.

## Conflict of Interest Statement

The authors declare that the research was conducted in the absence of any commercial or financial relationships that could be construed as a potential conflict of interest.

## References

[B1] Atkinson A. P. Dittrich W. H. Gemmell A. J. Young A. W. (2004). Emotion perception from dynamic and static body expressions in point-light and full-light displays. Perception 33, 717–746. 10.1068/p5096 15330366

[B62] Bates D. Maechler M. Bolker B. Walker S. Christensen R. H. B. Singmann H. (2014). lme4: Linear mixed-effects models using Eigen and S4.

[B2] Berkovich-Ohana A. Dor-Ziderman Y. Glicksohn J. Goldstein A. (2013). Alterations in the sense of time, space and body in the mindfulness-trained brain: a neurophenomenologically-guided MEG study. Front. Psychol. 4:912. 10.3389/fpsyg.2013.00912 24348455 PMC3847819

[B3] Berkovich-Ohana A. Glicksohn J. Goldstein A. (2011). Temporal cognition changes following mindfulness, but not transcendental meditation practice. Proc. Fechner Day 27, 245–250.

[B4] Boiten F. A. Frijda N. H. Wientjes C. J. E. (1994). Emotions and respiratory patterns: review and critical analysis. Int. J. Psychophysiol. 17, 103–128. 10.1016/0167-8760(94)90027-2 7995774

[B5] Brown S. Parsons L. M. (2008). The neuroscience of dance. Sci. Am. 299, 78–83. 10.1038/scientificamerican0708-78 18623968

[B6] Buccino G. Binkofski F. Fink G. R. Fadiga L. Fogassi L. Gallese V.. (2001). Action observation activates premotor and parietal areas in a somatotopic manner: an fMRI study. Eur. J. Neurosci. 13, 400–404. 10.1111/j.1460-9568.2001.01385.x 11168545

[B7] Cahoon R. L. (1969). Physiological arousal and time estimation. Percept. Mot. Skills 28, 259–268. 10.2466/pms.1969.28.1.259 5777952

[B9] Calvo-Merino B. Glaser D. E. Grèzes J. Passingham R. E. Haggard P. (2005). Action observation and acquired motor skills: an fMRI study with expert dancers. Cereb. Cortex 15, 1243–1249. 10.1093/cercor/bhi007 15616133

[B8] Calvo-Merino B. Grèzes J. Glaser D. E. Passingham R. E. Haggard P. (2006). Seeing or doing? Influence of visual and motor familiarity in action observation. Curr. Biol. 16, 1905–1910. 10.1016/j.cub.2006.07.065 17027486

[B10] Calvo-Merino B. Jola C. Glaser D. E. Haggard P. (2008). Towards a sensorimotor aesthetics of performing art. Conscious. Cogn. 17, 911–922. 10.1016/j.concog.2007.11.003 18207423

[B11] Cela-Conde C. J. Ayala F. J. Munar E. Maestú F. Nadal M. Capó M. A.. (2009). Sex-related similarities and differences in the neural correlates of beauty. Proc. Natl. Acad. Sci. U S A 106, 3847–3852. 10.1073/pnas.0900304106 19237562 PMC2656168

[B12] Chatterjee A. (2011). Neuroaesthetics: a coming of age story. J. Cogn. Neurosci. 23, 53–62. 10.1162/jocn.2010.21457 20175677

[B13] Clayton M. (2012). What is Entrainment? Definition and applications in musical research. Empir. Musicol. Rev. 7, 49–56.

[B14] Codrons E. Bernardi N. F. Vandoni M. Bernardi L. (2014). Spontaneous group synchronization of movements and respiratory rhythms. PLoS One 9:e107538. 10.1371/journal.pone.0107538 25216280 PMC4162643

[B15] Cross E. S. de C. Hamilton A. F. Grafton S. T. (2006). Building a motor simulation de novo: observation of dance by dancers. Neuroimage 31, 1257–1267. 10.1016/j.neuroimage.2006.01.033 16530429 PMC1821082

[B16] Cross E. S. Kirsch L. Ticini L. F. Schütz-Bosbach S. (2011). The impact of aesthetic evaluation and physical ability on dance perception. Front. Hum. Neurosci. 5:102. 10.3389/fnhum.2011.00102 21960969 PMC3177045

[B17] di Pellegrino G. Fadiga L. Fogassi L. Gallese V. Rizzolatti G. (1992). Understanding motor events: a neurophysiological study. Exp. Brain Res. 91, 176–180. 10.1007/bf00230027 1301372

[B18] Duncan S. Barrett L. F. (2007). Affect is a form of cognition: a neurobiological analysis. Cogn. Emot. 21, 1184–1211. 10.1080/02699930701437931 18509504 PMC2396787

[B19] Efron B. Morris C. (1977). Stein’s paradox in statistics. Sci. Am. 236, 119–127 10.1038/scientificamerican0577-119

[B20] Fadiga L. Fogassi L. Pavesi G. Rizzolatti G. (1995). Motor facilitation during action observation: a magnetic stimulation study. J. Neurophysiol. 73, 2608–2611. 7666169 10.1152/jn.1995.73.6.2608

[B21] Fontaine G. (2004). Les Danses Du Temps. ed. Centre National de la Danse, S.l.: S.n., 137.

[B22] Freedberg D. Gallese V. (2007). Motion, emotion and empathy in esthetic experience. Trends Cogn. Sci. 11, 197–203. 10.1016/j.tics.2007.02.003 17347026

[B23] Gibbon J. Church R. M. Meck W. H. (1984). Scalar timing in memory. Ann. N Y Acad. Sci. 423, 52–77. 10.1111/j.1749-6632.1984.tb23417.x 6588812

[B24] Gioffredi P. (2008). L’encadrement choré-graphique de l’expérience chez Myriam Gourfink, cadrages dans l’art des XXè et XXIè sièclesJournée d’étude du Centre de philosophie de l’art, Paris-1 Sorbonne, MSH Paris-Nord. Available online at: http://www.u-paris10.fr/15910355/0/fiche_pagelibre/&RH=1232798457967

[B25] Glicksohn J. (2001). Temporal cognition and the phenomenology of time: a multiplicative function for apparent duration. Conscious. Cogn. 10, 1–25. 10.1006/ccog.2000.0468 11273623

[B26] Grafton S. T. Hamilton A. F. D. C. (2007). Evidence for a distributed hierarchy of action representation in the brain. Hum. Mov. Sci. 26, 590–616. 10.1016/j.humov.2007.05.009 17706312 PMC2042582

[B27] Grommet E. K. Droit-Volet S. Gil S. Hemmes N. S. Baker A. H. Brown B. L. (2011). Time estimation of fear cues in human observers. Behav. Processes. 86, 88–93. 10.1016/j.beproc.2010.10.003 20971168

[B28] Haggard P. (2012). Talk at the The Conscious Body Workshop. Paris, France.

[B29] Hanna J. L. (1988). Dance, Sex and Gender: Signs of Identity, Dominance, Defiance and Desire. Chicago IL: University of Chicago Press.

[B30] Helm J. L. Sbarra D. Ferrer E. (2012). Assessing cross-partner associations in physiological responses via coupled oscillator models. Emotion 12, 748–762. 10.1037/a0025036 21910541

[B31] Hoagland H. (1933). The physiological control of judgments of duration: evidence for a chemical clock. J. Gen. Psychol. 9, 267–287 10.1080/00221309.1933.9920937

[B32] Hugill N. Fink B. Neave N. Seydel H. (2009). Men’s physical strength is associated with women’s perceptions of their dancing ability. Pers. Individ. Dif. 47, 527–530 10.1016/j.paid.2009.04.009

[B33] Jacobsen T. Schubotz R. I. Höfel L. Cramon D. Y. V. (2006). Brain correlates of aesthetic judgment of beauty. Neuroimage 29, 276–285. 10.1016/j.neuroimage.2005.07.010 16087351

[B34] Jeannerod M. (1999). The 25th Bartlett Lecture. To act or not to act: perspectives on the representation of actions. Q. J. Exp. Psychol. A 52, 1–29. 10.1080/713755803 10101973

[B35] Jola C. Abedian-Amiri A. Kuppuswamy A. Pollick F. E. Grosbras M.-H. (2012). Motor simulation without motor expertise: enhanced corticospinal excitability in visually experienced dance spectators. PLoS One 7:e33343. 10.1371/journal.pone.0033343 22457754 PMC3310063

[B36] Jola C. Grosbras M.-H. (2013). In the here and now: enhanced motor corticospinal excitability in novices when watching live compared to video recorded dance. Cogn. Neurosci. 4, 90–98. 10.1080/17588928.2013.776035 24073734

[B37] Kawabata H. Zeki S. (2004). Neural correlates of beauty. J. Neurophysiol. 91, 1699–1705. 10.1152/jn.00696.2003 15010496

[B38] Knoblich G. Sebanz N. (2008). Evolving intentions for social interaction: from entrainment to joint action. Philos. Trans. R. Soc. Lond. B Biol. Sci. 363, 2021–2031. 10.1098/rstb.2008.0006 18292061 PMC2606699

[B39] Konvalinka I. Xygalatas D. Bulbulia J. Schjødt U. Jegindø E.-M. Wallot S.. (2011). Synchronized arousal between performers and related spectators in a fire-walking ritual. Proc. Natl. Acad. Sci. U S A 108, 8514–8519. 10.1073/pnas.1016955108 21536887 PMC3100954

[B40] Kramer R. Weger U. Sharma D. (2013). The effect of mindfulness meditation on time perception. Conscious. Cogn. 22, 846–852. 10.1016/j.concog.2013.05.008 23778017

[B41] Lamotte M. Izaute M. Droit-Volet S. (2012). Awareness of time distortions and its relation with time judgment: a metacognitive approach. Conscious. Cogn. 21, 835–842. 10.1016/j.concog.2012.02.012 22429850

[B42] Latulipe C. Carroll E. A. Lottridge D. (2011). “Love, hate, arousal and engagement: exploring audience responses to performing arts,” in Proceedings of the SIGCHI Conference on Human Factors in Computing Systems (Vancouver BC, Canada: ACM).

[B43] Leder H. Bär S. Topolinski S. (2012). Covert painting simulations influence aesthetic appreciation of artworks. Psychol. Sci. 23, 1479–1481. 10.1177/0956797612452866 23137968

[B44] Lesauvage M. Piettre C. (eds) (2011). Myriam Gourfink, Danser Sa Créature. Dijon: Presses du réel.

[B45] Macel C. (2008). Le Temps Pris: Le Temps de L’oeuvre, Le Temps à L’oeuvre. Paris: Monografik; Centre Pompidou, Blou.

[B46] Nadal M. Munar E. Capó M. A. Rosselló J. Cela-Conde C. J. (2008). Towards a framework for the study of the neural correlates of aesthetic preference. Spat. Vis. 21, 379–396. 10.1163/156856808784532653 18534110

[B47] Nather F. C. Bueno J. L. O. Bigand E. Droit-Volet S. (2011). Time changes with the embodiment of another’s body posture. PLoS One 6:e19818. 10.1371/journal.pone.0019818 21637759 PMC3103514

[B48] Orgs G. Bestmann S. Schuur F. Haggard P. (2011). From body form to biological motion: the apparent velocity of human movement biases subjective time. Psychol. Sci. 22, 712–717. 10.1177/0956797611406446 21525378 PMC3693441

[B49] Pollatos O. Laubrock J. Wittmann M. (2014). Interoceptive focus shapes the experience of time. PLoS One 9:e86934. 10.1371/journal.pone.0086934 24489807 PMC3906083

[B50] Richardson M. J. Marsh K. L. Isenhower R. W. Goodman J. R. L. Schmidt R. C. (2007). Rocking together: dynamics of intentional and unintentional interpersonal coordination. Hum. Mov. Sci. 26, 867–891. 10.1016/j.humov.2007.07.002 17765345

[B51] Rickard N. S. (2004). Intense emotional responses to music: a test of the physiological arousal hypothesis. Psychol. Music 32, 371–388 10.1177/0305735604046096

[B52] Rizzolatti G. Fadiga L. Gallese V. Fogassi L. (1996). Premotor cortex and the recognition of motor actions. Brain Res. Cogn. Brain Res. 3, 131–141. 10.1016/0926-6410(95)00038-0 8713554

[B53] Sauer S. Lemke J. Wittmann M. Kohls N. Mochty U. Walach H. (2012). How long is now for mindfulness meditators? Pers. Individ. Dif. 52, 750–754 10.1016/j.paid.2011.12.026

[B54] Schwartz W. (1978). Time and context during hypnotic involvement. Int. J. Clin. Exp. Hypn. 26, 307–316. 10.1080/00207147808411255 681032

[B55] Sgouramani H. Vatakis A. (2014). “Flash” dance: how speed modulates perceived duration in dancers and non-dancers. Acta Psychol. (Amst) 147, 17–24. 10.1016/j.actpsy.2013.06.009 23910150

[B56] Sperry R. W. (1952). Neurology and the mind-brain problem. Am. Sci. 40, 291–312.

[B57] Stevens J. A. Fonlupt P. Shiffrar M. Decety J. (2000). New aspects of motion perception: selective neural encoding of apparent human movements. Neuroreport 11, 109–115. 10.1097/00001756-200001170-00022 10683840

[B58] van Baaren R. B. Holland R. W. Kawakami K. van Knippenberg A. (2004). Mimicry and prosocial behavior. Psychol. Sci. 15, 71–74. 10.1111/j.0963-7214.2004.01501012.x 14717835

[B59] Varela F. J. (1996). Neurophenomenology: a methodological remedy for the hard problem. J. Conscious. Stud. 3, 330–349.

[B60] Wittmann M. Otten S. Schötz E. Sarikaya A. Lehnen H. Jo H.. (2015). Subjective expansion of extended time-spans in experienced meditators. Front. Psychol. 5:1586. 10.3389/fpsyg.2014.01586 25642205 PMC4294119

[B61] Wittmann M. van Wassenhove V. (2009). The experience of time: neural mechanisms and the interplay of emotion, cognition and embodiment. Philos. Trans. R. Soc. Lond. B Biol. Sci. 364, 1809–1813. 10.1098/rstb.2009.0025 19487184 PMC2685824

